# Cross-sectional analysis of *Piroplasma* species-infecting camel (*Camelus dromedaries*) in Egypt using a multipronged molecular diagnostic approach

**DOI:** 10.3389/fvets.2023.1178511

**Published:** 2023-04-28

**Authors:** Olfat A. Mahdy, Ahmed M. Nassar, Bassma S. M. Elsawy, Heba F. Alzan, Omnia M. Kandil, Mona S. Mahmoud, Carlos E. Suarez

**Affiliations:** ^1^Parasitology Department, Faculty of Veterinary Medicine, Cairo University, Giza, Egypt; ^2^Parasitology and Animal Diseases Department, Veterinary Institute, National Research Centre, Giza, Egypt; ^3^Tick and Tick-Borne Diseases Research Unit, National Research Centre, Giza, Egypt; ^4^Department of Veterinary Microbiology and Pathology, College of Veterinary Medicine, Washington State University, Pullman, WA, United States; ^5^Department of Agricultural - Agricultural Research Service, Pullman, WA, United States

**Keywords:** *Piroplasma* spp, camel, genetic characterization, phylogenetic analysis, Egypt

## Abstract

Camel piroplasmosis is a tick-borne disease (TBD) caused by hemoprotozoan parasites. Hereby, we describe a cross-sectional study aiming at identifying *Piroplasma* spp.-infecting camels in Egypt using a multipronged molecular diagnostic approach. A total of 531 blood samples from camels (*Camelus dromedarius*) were collected from slaughterhouses at different governorates in Egypt for analysis during the period from June 2018 to May 2019. *Piroplasma* spp. was identified using microscopical examination and several different and sequential polymerase chain reaction (PCR) assays targeting the 18S rRNA genes. The overall prevalence of *Piroplasma* spp. in microscopical and molecular analyses in the samples was 11% (58/531) and 38% (203/531), respectively. Further discriminative multiplex PCR analysis targeting the 18S rRNA gene applied on all *Piroplasma* spp.-positive samples allowed the detection of *Theileria equi* (41%)*, Babesia caballi* (5.4%)*, Babesia bigemina* (0.5%), and *Babesia bovis* (4%). Additionally, the blast analysis of nested (n) PCR, targeting the V4 region, amplicon sequences resulted in the identification of *B. vulpes* (22%), *Babesia* sp. (9%), and *Theileria* sp. (3%). Overall, the results of this study confirmed the high prevalence of TBDs caused by several types of piroplasm hemoparasites in camel and suggests the need for future interventions aimed at improving the control of these potentially debilitating diseases that may be t-hreatening important economic resources and food security in Egypt.

## 1. Introduction

Camel is a multipurpose animal widely distributed in Africa, the Middle East, and northern India that has been utilized for food and recreational purposes (1, 2). There is a steady increase in the number of camels slaughtered for meat in many developing countries, including Egypt (1). Sudan and Ethiopia are the main sources of camels for Egypt, with more than 750,000 camels imported between 2012 and 2015 (2).

Camel piroplasmosis is an infectious disease of camel distributed worldwide and caused by hemoprotozoan piroplasmid parasites, which belong to the phylum Apicomplexa. The disease is characterized by high morbidity and mortality, especially if untreated, and is responsible for substantial economic losses (3, 4). Clinical manifestations include anemia, hemoglobinuria, muscle trembling, and a decrease in body temperature to subnormal levels (3, 5). Piroplasmosis in camel is understudied and known to be caused by hemoparasites, such as *Theileria* (T.) *equi, Babesia* (B.) *caballi* (6–9), *Babesia bovis, Babesia bigemina* (3, 10), and *Theileria camelensis* (11). However, the taxonomic status of *T. camelensis* remains unclear due to a lack of studies involving experimental infections and molecular characterization (12). In addition *Theileria annulata* and *Theileria ovis* (2, 12, 13) were also reported to infect camel, so the vertebrate host-specificity of these tick-borne blood parasites is probably wider than expected (12). Ticks play a critical role in the transmission of these parasites as the definitive host of these apicomplexan organisms (9). In Egypt, camels are known to be infested by different species of ticks, including *Hyalomma* (H.) *dromedarii, Hyalomma rufipes, Hyalomma truncatum, Hyalomma anatolicum excavatum*, and *Hyalomma impeltatum*, in addition to *Rhipicephalus* (R.) *annulatus, Rhipicephalus sanguineus* “*sensu lato*” (14), *Rhipicephalus pulchellus, Amblyomma* (A.) *gemma, Amblyomma lepidum*, and *Amblyomma variegatum* (15). Importantly, some of these tick species are known to be competent vectors of piroplasmid parasites. Altogether, the presence of competent ticks and *Piroplasma*-infected camels, which may act as parasite reservoirs, may pose an important risk factor for farm animals, such as cattle and sheep, residing in endemic areas.

Previous studies addressing camel piroplasmosis were mostly based on the identification of piroplasmid species using microscopical examination or polymerase chain reaction (PCR) without performing sequencing and phylogenetic analysis (6–10, 16). However, the single use of microscopical examination for identifying piroplasm lacks specificity and can be prone to false negative results, especially in carrier animals with low parasitemia. In addition, identification using microscopy is limited at the genus level. In contrast, PCR techniques allow for the detection of DNAs of blood parasites with higher sensitivity and specificity (10, 17). Therefore, sensitive diagnostic tools, such as PCR-based assays such as uniplex PCR (uPCR) and multiplex PCR (mPCR), accompanied by amplicon sequencing, are better suited to state-of-the-art techniques for identifying *Piroplasma* species that infect camel (18). Uniplex PCR (uPCR) is a useful diagnostic tool in those cases involving a single infective agent, but it is considered time-consuming and expensive when mixed infections are present, especially when applied to many samples (19). In contrast, multiplex PCR (mPCR) assays are effective alternative tools that can be used for the simultaneous detection of multiple specific species of tick-borne parasitic diseases (TBDs) in clinical samples (20).

In this study, we describe the identification and estimation of the prevalence of piroplasm parasites infecting camels from different locations in Egypt using a multipronged PCR-based approach. Phylogenetic analysis of the identified piroplasm species was also performed using sequences of amplicons derived from a conserved region in 18S rRNA of *Babesia*/*Theileria* spp. More specifically, this analysis was performed using universal primers targeting the hypervariable region (V4) in 18S of *Piroplasma* spp. in nested PCR (nPCR), followed by sequencing for identifying piroplasm species-infecting camel.

## 2. Materials and methods

### 2.1. Collection of field camel blood samples

**Figure 1:**
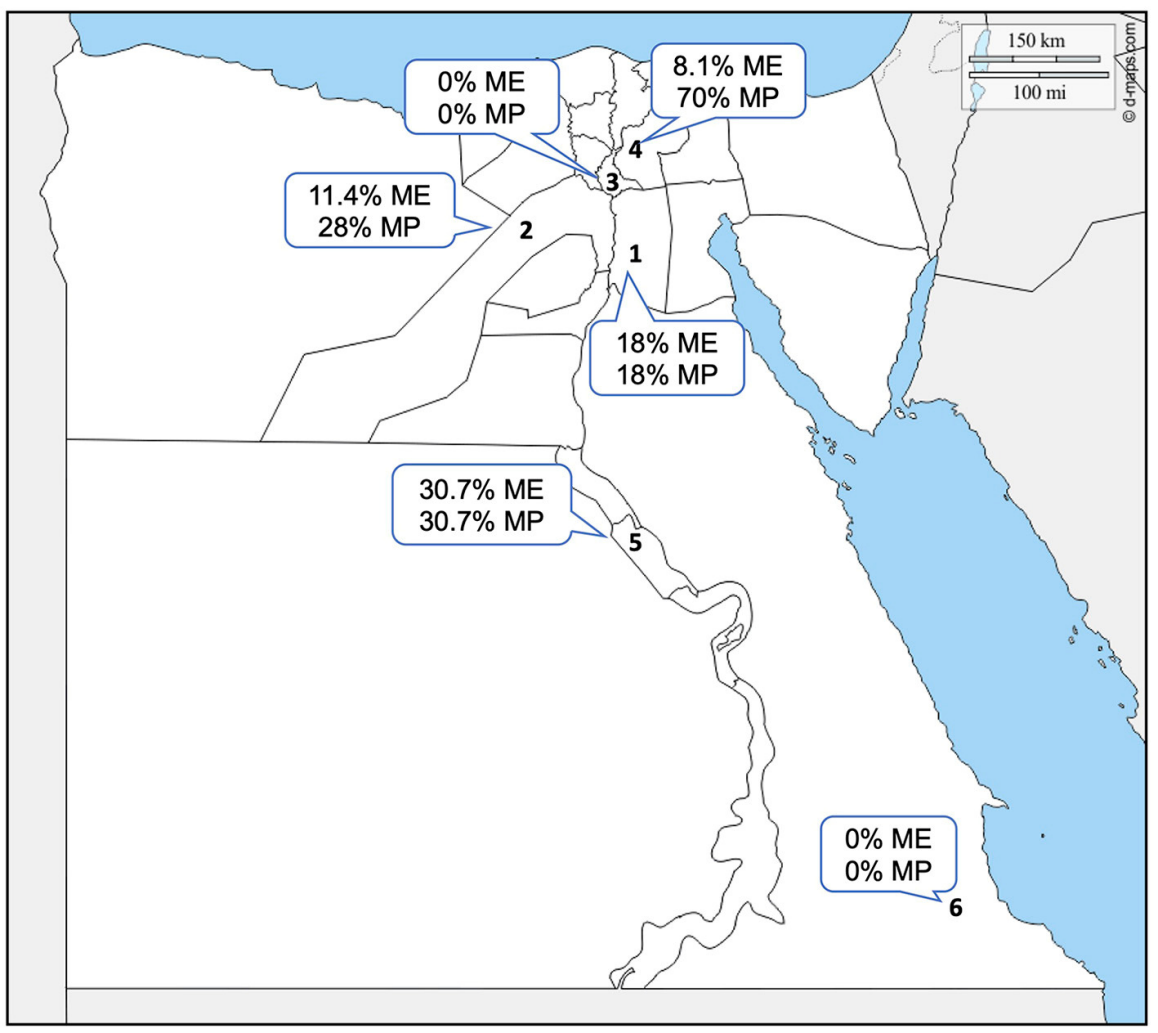
Geographic location of the sampling sites in Egypt. Arabic numbers represent the governorates in the study that were used for sampling. 1. Cairo, 2. Giza, 3. Qalubya, 4. Sharkia, 5. Suhag, and 6. Halayb w Shalatin. ME, microscopical examination; MP, molecular prevalence.

Blood samples were collected in tubes containing EDTA from each of the 531 slaughtered camels during the period from June 2018 to May 2019. The blood samples were collected from slaughterhouses from different governorates in Egypt (Cairo 30°2′0″N, 31°14′0″E, Giza 29°59′13.2″N, 31°12′42.48″E, Qalubya 30.41°N 31.21°E, Sharika 30.7°N 31.63°E, Suhag 26.56°N 31.7°E, and Halayb w Shalatin 25°32′1″N 33°26′18″E), as described in Figure 1. The blood smears were made immediately; then, the rest of the blood samples were transferred in refrigerated boxes to the National Research Centre (NRC) laboratory, Dokki, Egypt, for DNA extraction.

### 2.2. Parasitological examination

#### 2.2.1. Blood film examination

Thin blood films were prepared from each of the 531 blood samples and stained with Diff-3 stain according to the manufacturer's instructions (ILT Diagnostic, Egypt). The stained blood smears were examined under an oil immersion lens of a light microscope (Zeiss, Germany) to identify infected animals and study the morphological characteristics of infecting parasites.

#### 2.2.2. Molecular detection

##### 2.2.2.1. DNA extraction

Genomic DNA was extracted from the whole blood of 531 camel samples by using DNA extraction kits (QIAamp DNA Blood Mini Kit, QIAGEN/Germany), following the manufacturer's instructions. Positive control *T. equi, B. caballi, B. bovis*, and *B. bigemina* DNA were kindly donated from Animal Disease Research Unit located in Pullman, WA, United States.

##### 2.2.2.2. Conventional PCR method for the detection of camel piroplasmosis

Conventional PCR (cPCR) was used as a preliminary survey for the detection of the prevalence of *Piroplasma* species-infecting camel. A total of 531 DNA samples were tested by cPCR using universal primers (TB-F&TB-R; Table 1), to amplify a fragment of *Babesia*/*Theileria* spp. 18S rRNA gene using identical PCR conditions as previously described (21). In brief, the reaction conditions in 25 μl volume were composed of 3 μl of the DNA sample, 12.5 μl of master mix, 1 μl of each primer, and 7.5 μl of nuclease-free water. The amplification conditions were as follows: 5 min at 94°C, 40 cycles each of 94°C for 45 s, 61°C for 30 s, and 72°C for 45 s, in addition to the final extension period for 7 min at 72°C. *Babesia* and *Theileria* spp. DNA were included in each PCR reaction as positive controls. Negative controls lacking DNA were also included. Products of all PCR reactions were subjected to SYBR Safe DNA gel staining (Invitrogen, Waltham, United States) on 1.5% agarose gel electrophoresis (Invitrogen, Waltham, United States). The length of the amplified products was estimated by comparing them with a 1 kbp plus DNA ladder (Invitrogen, United States), and the amplified products were visualized using a gel documentation system (Bio-Rad, California, United States).

**Table 1:** Oligonucleotide primers used in conventional and multiplex PCR.

**Parasite**	**Gene**	**PCR**	**Size**	**Primer forward**	**Primer reverse**	**References**
*Piroplasma*	18S rRNA	cPCR	496 bp	5′CTTCAGCACCTTGAGAGAAAT3′	5′TCDATCCCCRWCACGATGCRBAC3′	(21)
*B. caballi*	18S rRNA	mPCR	540 bp	5′TCGAAGACGATCAGATACCGTCG3′	5′CTCGTTCATGATTTAGAATTGCT3′	(23)
*T. equi*	18S rRNA	mPCR	360 bp	5′CT TCAGCACCTTGAGAGAAATC3′	5′TGCCTTAAACTTCCTTGCGAT3′	(8)
*B. bovis*	CPSII	mPCR	448 bp	5′TCGAAGACGATCAGATACCGTCG3′	5′ACCACTGTAGTCAAACTCACC3′	(22)
*B. bigemina*	18S rRNA	mPCR	1,124 bp	5′CT TCAGCACCTTGAGAGAAATC3′	5′TGCCTTAAACTTCCTTGCGAT3′	(8)

### 2.3. Molecular characterization of piroplasm infecting camel

#### 2.3.1. Multiplex PCR

Multiplex PCR (mPCR) was performed on the DNA samples that were verified to be positive by cPCR, performed as described in the previous section, using specific primers for the amplification of amplicons from *T. equi, B. caballi*, and *B. bigemina* 18S rRNA genes and the *B. bovis* carbamoyl phosphate synthetase (CPSII) gene. The CPSII gene is highly conserved and encodes for an enzyme responsible for *de novo* pyrimidine biosynthesis (22). Primer sequences used in mPCR are shown in Table 1. The mPCR reactions were performed according to Alhassan et al. (23). In brief, the reactions were accomplished in volumes of 25 μl PCR reaction which consisted of 3.5 μl of the DNA sample, 12.5 μl of Master Mix (Genedirx), 2.5 pmol of each of the primers, and 7 μl nuclease-free water; amplification conditions were as follows: 5 min at 94°C, 35 cycles each of 94°C for 1 min, 61°C for 1 min, and 72°C for 1 min, with the addition of a final extension period of 7 min at 72°C. The mPCR-positive samples were further screened and confirmed using each species-specific primer separately by uniplex PCR (uPCR) (Table 1) and sequenced. Amplicons that showed strong positive by uPCR were purified using Thermo scientific gene JET gel extraction kits and sent for bi-directional Sanger sequencing using the ABI3730XL DNA sequencer (Macrogen Inc., South Korea). Species-specific sequencing primers are presented in Table 2.

**Table 2:** Oligonucleotide primers used for DNA sequencing.

**Parasite**	**Gene**	**PCR**	**Size**	**Primer forward**	**Primer reverse**	**References**
*Piroplasma*	18S rRNA	nPCR	390	RLB-F2: 5′GACACAGGGAGGTAGTGACAAG3′	RLBR2: 5′CTAAGAATTTCACCTCTGACAGT3′	(24)
			388	FINT: 5′GACAAGAAATAACAATACRGGGC3′		
*B. caballi*	18S rRNA	uPCR	540	5′TCG AAG ACG ATC AGA TAC CGT CG3′	5′CTCGTTCATGATTTAGAATTG CT3′	(23)
*T. equi*	18S rRNA	uPCR	360	5′CTTCAGCACCTTGAGAGAAATC3′	5′TGCCTTAAACTTCCTTGCGAT3′	(8)
*B. bovis*	CPSII	uPCR	448	5′TTTGGTATTTGTCTT GGTCAT3′	5′ACCACTGTAGTCAAACTCACC3′	(22)
*B. bigemina*	18S rRNA	uPCR	639	5′TAGTTGTATTTCAGCCTCGCG3′	5′AACATCCAAGCAGCTAHTTAG3′	(10)

#### 2.3.2. Nested PCR targeting the V4 region of the 18S rRNA gene to detect Piroplasma spp.-infecting camels

A group of purified DNA samples that tested positive for *Piroplasma* spp. by cPCR and negative by mPCR was further screened by nPCR using primers targeting the hypervariable (V4) region of the 18S rRNA gene. The primer sequences were used for external PCR reaction [RLB-F2 and RLB-R2] and nPCR [RLB-FINT and RLB-R2]. The PCR external and internal reaction conditions were set up according to Liu et al. (24). The PCR products obtained from representative positive samples by nPCR were purified using ExoSAP-IT reagent (Applied Biosystems, Lithuania, North-eastern Europe) and then sequenced (Sanger sequencing method, Eurofins Genomics, SimpleSeq service, Louisville, KY, United States).

### 2.4. Sequence analysis

The basic local alignment search tool (BLAST) was used for species identification of DNA sequences. The uPCR and nPCR sample sequencing results were aligned with each species reference sequence and edited using MEGA7 software (https://www.megasoftware.net/download_form). Query coverage and the identity percentage among the compared sequences were calculated by NCBI and Clustal Omega website (https://blast.ncbi.nlm.nih.gov/Blast.cgi and https://www.ebi.ac.uk/Tools/msa/clustalo/). All sequences of the *Piroplasma* spp. Egyptian isolates in camel were assigned accession numbers upon submission to GenBank.

### 2.5. Phylogenetic analysis

Piroplasm genetic diversity was assessed by constructing dendrograms using phylogenetic tree prediction generated by MEGA7 (https://www.megasoftware.net/download_form). This dendrogram was constructed using the Maximum Likelihood method based on the Kimura 2-parameter mode (25). The sequences obtained from the Egyptian camel piroplasm and different reference sequences in GenBank were used for comparative molecular analysis. *Eimeria* sp. cytochrome oxidase subunit I (COI) gene (KT305929.1) (26) and *B. bovis* (AY150059.1) (27) were included in the trees as outgroups.

### 2.6. Statistical analysis

A chi-square (χ^2^) test was applied at a probability of *P* < 0.05 to compare the camel infection rates in different seasons using cPCR. In addition, the confidence interval (CI) was calculated according to Bevans (28).

## 3. Results

### 3.1. Parasitological examination

#### 3.1.1. Detection of Piroplasma species in camel blood smear samples

**Figure 2:**
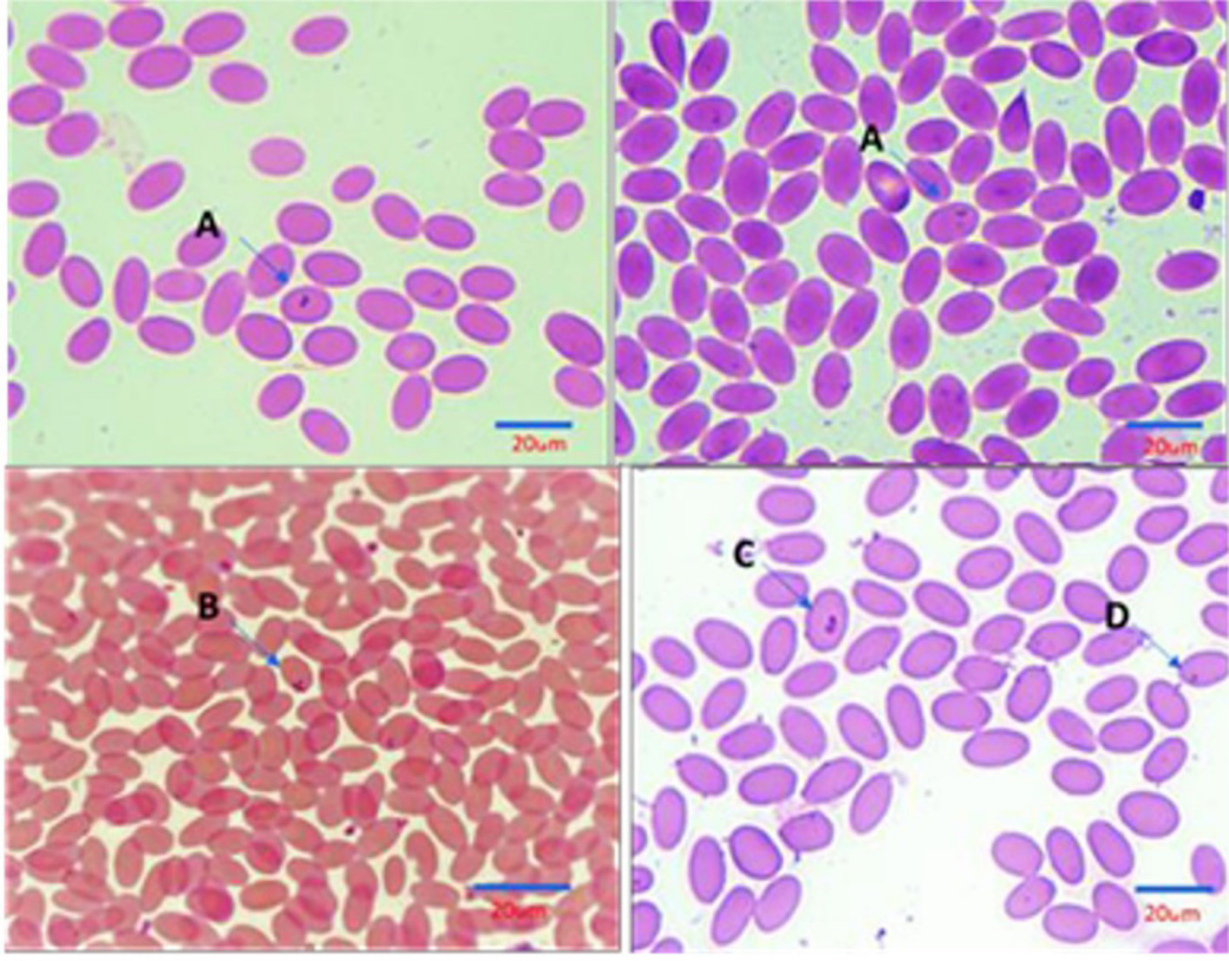
Illustration of Intra erythrocytic stages of *Piroplasma*-infecting camel under the light microscope (Diff Quick, 1,000 ×). **(A)** Double small piroplasmid; **(B)** Maltese cross formation; **(C)** Double large piroplasmid; **(D)** Single small piroplasm (1.5–2 μm). Size bars represent 20 mm.

Examination of stained camel blood smears revealed the presence of intraerythrocytic pleomorphic pear or oval shapes of piroplasmid including the following: [A]: double small piroplasm measuring 1.5–2 μm; [B]: maltese cross) four pear-shaped merozoites; each merozoite measures approximately 2 μm in length); [C]: double large piroplasm measuring 3–5 μm; and [D]: single small piroplasm measuring 1.5–2 μm (representative images for each form are shown in Figure 2).

#### 3.1.2. Prevalence of Piroplasma spp. infections in camels: comparison among microscopic and conventional PCR methods

##### 3.1.2.1. Microscopical prevalence

Microscopical examination of Diff–Quick-stained blood smears revealed that 58 (11%) of 531 camel samples under analysis were infected with *Piroplasma* spp., as presented in Table 3 [95% confidence interval (CI) 8–14%]. The microscopical prevalence in each locality was 18% in Cairo, 11.4% in Giza, 0% in Qalubia, 8.1% in Sharkia, 30.7% in Suhag, and 0% in Halayb w Shalatin (Figure 1).

**Table 3:** Parasitological and molecular prevalence of *Piroplasma* spp. infections in camel.

**Total no. of examined samples**	**Positive samples by microscopy No. (%, 95%CI)**	**Positive samples by cPCR No. (%, 95% CI)**
531	58 (11%, 95%CI, 8–14%)	203 (38%, 95% CI, 34–42%)
Chi^2^	14.8	
*P* value	0.000	

No, number; 95% CI, 95% confidence interval.

##### 3.1.2.2. Molecular prevalence of *Piroplasma* spp. by conventional PCR

**Figure 3:**
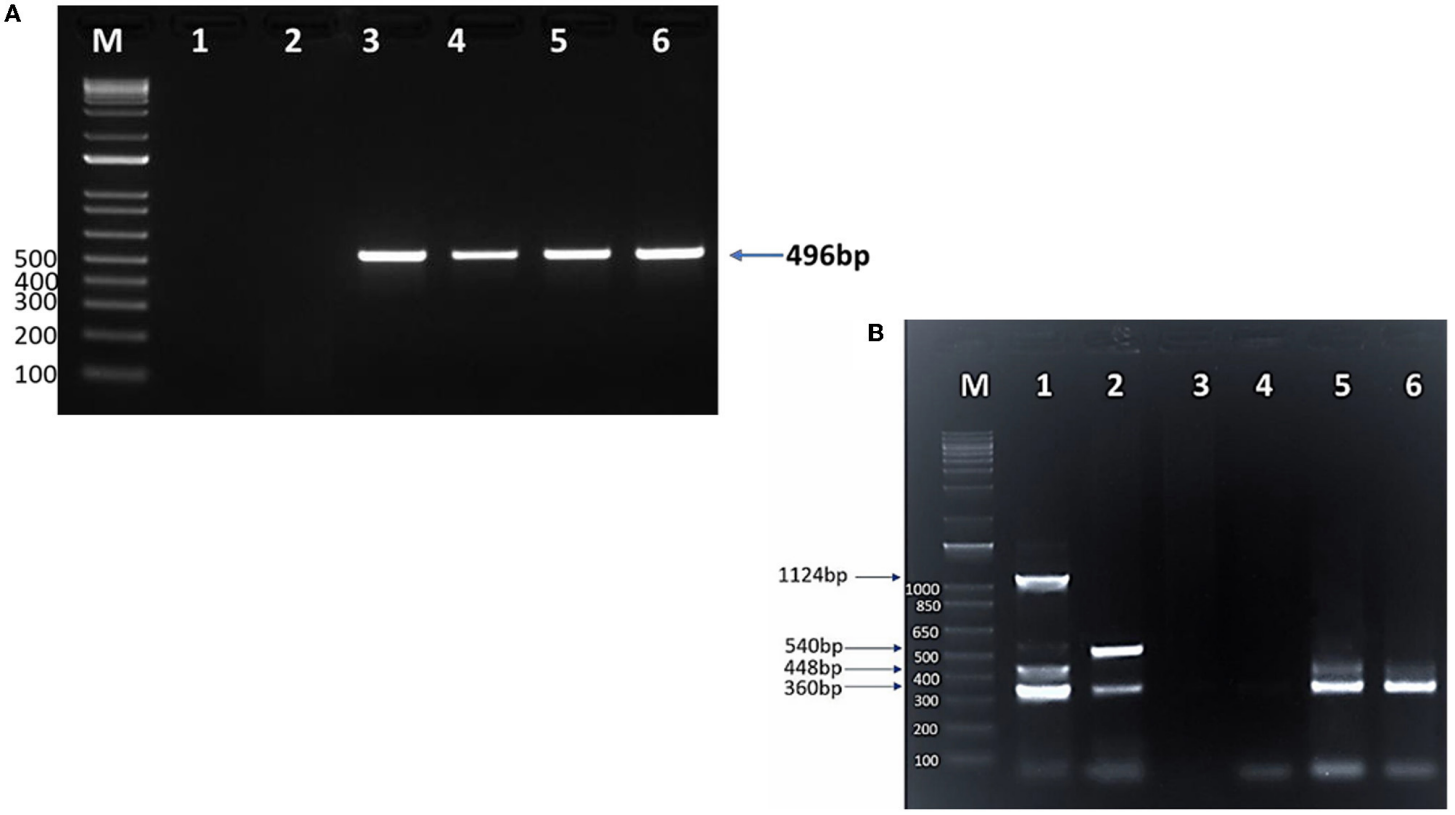
SYPR safe stained 1.5% agarose gel electrophoresis of **(A)** cPCR products of *Piroplasma* spp. M: 1 Kb DNA ladder; lane 1: negative control; lane 2: negative camel DNA sample; lane 3: *Piroplasma* spp. positive control (496 bp); lanes 4, 5, and 6: positive *Piroplasma* spp. camel DNA samples. **(B)** Multiplex PCR for detection of *T. equi, B. bovis, B. caballi*, and *B. bigemina* in camels. M: 1 Kb DNA ladder; lane 1: *T. equi* (360 bp), *B. bovis* (448 bp), *B. caballi* (540 bp), and *B. bigemina* (1,124 bp) positive controls DNA; lane 2: mixed infection with *T. equi* and *B. caballi*; lane 3: negative control (sterile H_2_O instead of DNA); lane 4: negative DNA sample; lane 5: mixed infection with *T. equi* and *B. bovis*; lane 6: *T. equi* single infection.

Conventional PCR (cPCR) using universal primers targeting a pan-piroplasm conserved region of the18S gene detected *Babesia/Theileria* spp. DNA in 203 (38%; 95%CI: 34–42%) out of 531 camel DNA samples (Table 3) with the expected 496 bp amplicon size (Figure 3A). All blood samples derived from the 58 camel samples that were found positive by microscopy turned out positive in the cPCR analysis.

The molecular prevalence in each locality was 18% in Cairo, 28% in Giza, 0% in Qalubia, 70% in Sharkia, and 30.7% in Suhag and Halayb w Shalatin (Figure 1).

### 3.2. Molecular identification of *Piroplasma* spp-infecting camel

#### 3.2.1. Multiplex PCR for simultaneous detection of Piroplasma spp.

The mPCR was performed on the cohort of 203 DNA *Piroplasma* spp. cPCR-positive samples. The mPCR analysis was devised to detect *T. equi, B. caballi, B. bigemina*, and *B. bovis* DNA, which generate amplicons with expected sizes of 360, 540, 1,124, and 448 bp, respectively (Figure 3B). The results of this experiment are presented in Table 4. The mPCR was unable to generate amplicons in 87 out of the 203 samples but detected 116 positive samples, as described in Table 4. The highest incidence was registered for *T. equi* with single infections detected in 84 (41%, 95% CI, 34–48%) camel samples. Co-infections of *T. equi* with *B. caballi* were detected in 11 (5.4%, 95% CI, 2–8%) samples, and mixed infections of *T. equi* with *B. bovis* were detected in 10 (5%, 95% CI, 2–8%) samples. In addition, camel (0.5%, 95% CI, 0.5–1.5%) infected with *T. equi* was found also to be co-infected with *B. bovis* and *B. bigemina*. Moreover, camels infected with *B. bovis* single infection were detected in nine samples (4%, 95% CI, 1–7%). Mixed infection of *B. bovis* with *B. bigemina* was detected in only one sample (0.5%, 95% CI, 0.5–1.5%), as shown in Table 4 and Figure 3B. The mPCR was, then, followed by confirmatory uPCR analysis which was only performed in samples that were strongly positive (*n* = 16). All 16 samples were also sequenced, as described in the section below. In addition, the 87 samples that resulted negative in mPCR were further analyzed by nPCR, as described in the following section.

**Table 4:** Results of mPCR using *Theileria equi, Babesia caballi, Babesia bovis*, and *Babesia bigemina* specific primers.

**Number of positive camel samples by cPCR**	**Number of negative camel samples by mPCR**	**Single infection**				**Mixed infection**							
		***T. equi***		***B. bovis***		***T. equi*** **with** ***B. caballi***		***T. equi*** **with** ***B. bovis***		***T. equi*** **with** ***B.bovis*** **and** ***B. bigemina***		***B.bovis with B. bigemina***	
		**N (%)**	**(95% CI)**	**N (%)**	**(95%CI)**	**N (%)**	**(95%CI)**	**N (%)**	**(95%CI)**	**N (%)**	**(95%CI)**	**N (%)**	**(95%CI)**
203	87	84 (41%)	(34–48%)	9 (4%)	(1–7%)	11 (5.4%)	(2–8%)	10 (5%)	(2–8%)	One sample (0.5%)	(0.5–1.5%)	One sample (0.5%)	(0.5–1.5%)

N, number of camels.

### 3.3. Detection of *Piroplasma* spp. infecting camel using nPCR

An nPCR using primers targeting 388 bp of the V4 region of the 18S gene was applied on 87 out of the 203 samples that tested positive by cPCR but negative for mPCR. We took this approach since the mPCR was targeting only four hemoparasite spp. and has lower sensitivity than nPCR. Using this method, we detected amplicons in all 87 analyzed samples. Sequencing analysis of 33 selected representative nPCR-positive samples revealed that the prevalence of *B. vulpes* in camels was 22% (seven samples), *Babesia* sp. was 9% (four samples), *Theileria* sp. was 3% (one sample), *T. equi* was 16% (five samples), and *B. caballi* was 50% (16 samples).

**Figure 4:**
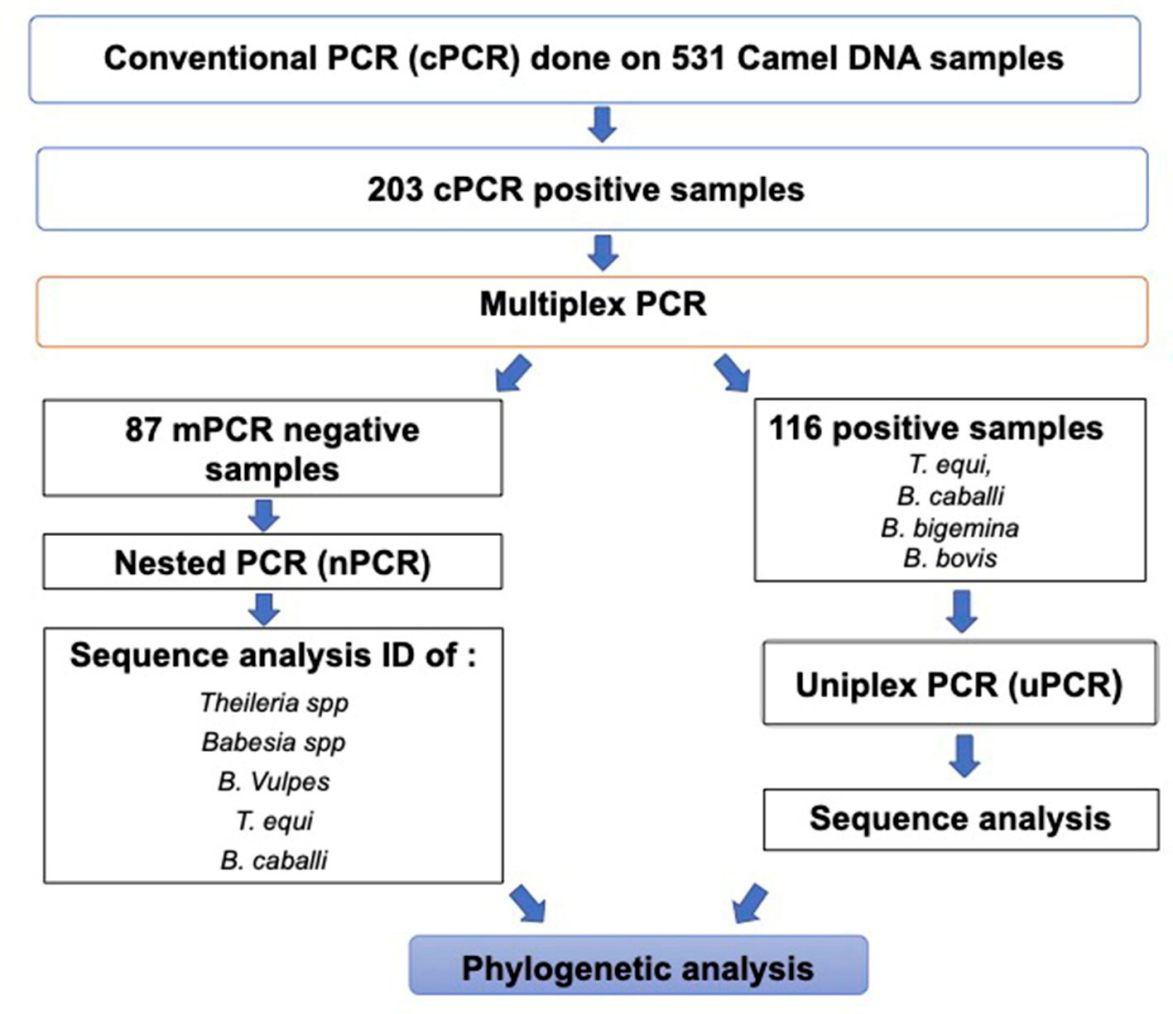
Workflow chart describing the molecular diagnostic procedures and a summary of the molecular diagnostic data in this study.

A summary of the workflow and results of the molecular diagnostic work performed in this study is shown in Figure 4.

### 3.4. Comparative DNA sequence analysis of the *Piroplasma* spp. amplicons derived from camels by mPCR

All uPCR amplicons were submitted for sequencing (Figure 4). The resulted sequencing data were used to confirm the identity of the infective agent and perform phylogenetic analysis (Figure 4). All sequences were, then, analyzed by nBLAST to verify their identity upon sequence comparisons with sequences currently deposited in the NCBI database. Blast analysis indicated that 10 selected *T. equi*-positive samples were derived from camels in Egypt in this study by uPCR, with accession no. MZ562708.1 to MZ562717.1, showed identity between 98.1% and 100% with 100% query coverage to previously published sequences of the 18S rRNA gene of *T. equi* isolates from equines from Chile, China, and Israel. In addition, the *B. caballi* camel isolates (*n* = 2) with accession no. MZ675521.1–MZ675522.1 showed a percent of identities ranging from 99.5 to 100% with 100% query coverage to *B. caballi* isolates from Iraq, Turkey, and India. The presumptive *B. bigemina* sequence (*n* = 1) amplified from a camel with accession no. MZ675519.1 is 100% identical, with query coverage 100%, to sequences derived from *B. bigemina* isolates from cattle from the USA, South Africa, Colombia, and India. In addition, *B. bovis* camel-derived sequences (*n* = 3) with accession no. OK086022.1 to OK086024.1 had a 100% identity with 97.1–97.6% query coverage with *B. bovis* CPSII gene isolates from Iraq (camel origin). The percentages of identity among *T. equi, B. caballi, B. bigemina*, and *B. bovis* camel-derived sequences from Egypt and other countries are shown in Supplementary Tables 1–4.

Further phylogenetic analysis showed that camel *T. equi* sequences, with accession no. MZ562708 to MZ562717, clustered with equine *T. equi* sequences from Chile, Israel, China, Turkey, and the Kingdom of Saudi Arabia (KSA) as demonstrated in Supplementary Figure 1A. Similarly, camel-derived *B. caballi* sequences in the present study with accession no. MZ675521.1–MZ675522.1 clustered with equine *B. caballi* sequences from Egypt, Iraq, Turkey, and India (Supplementary Figure 1B).

In addition, phylogenetic analysis of the *B. bigemina* camel-derived sequence with the sequence identified by accession no. MZ675519.1 showed clustering with bovine-derived *B. bigemina* sequences from South Africa, Colombia, and the US (Supplementary Figure 1C). Finally, the *B. bovis* camel-derived sequences (*n* = 3) identified in the present study with accession no. OK086022.1–OK086024.1 clustered with camel *B. bovis* sequences from Iraq and bovine *B. bovis* sequences from the US and Australia (Supplementary Figure 2).

### 3.5. Phylogenetic analysis of *B. vulpes, Babesia* sp., and *Theileria* sp. camel-derived sequences from Egyptian isolates

**Figure 5:**
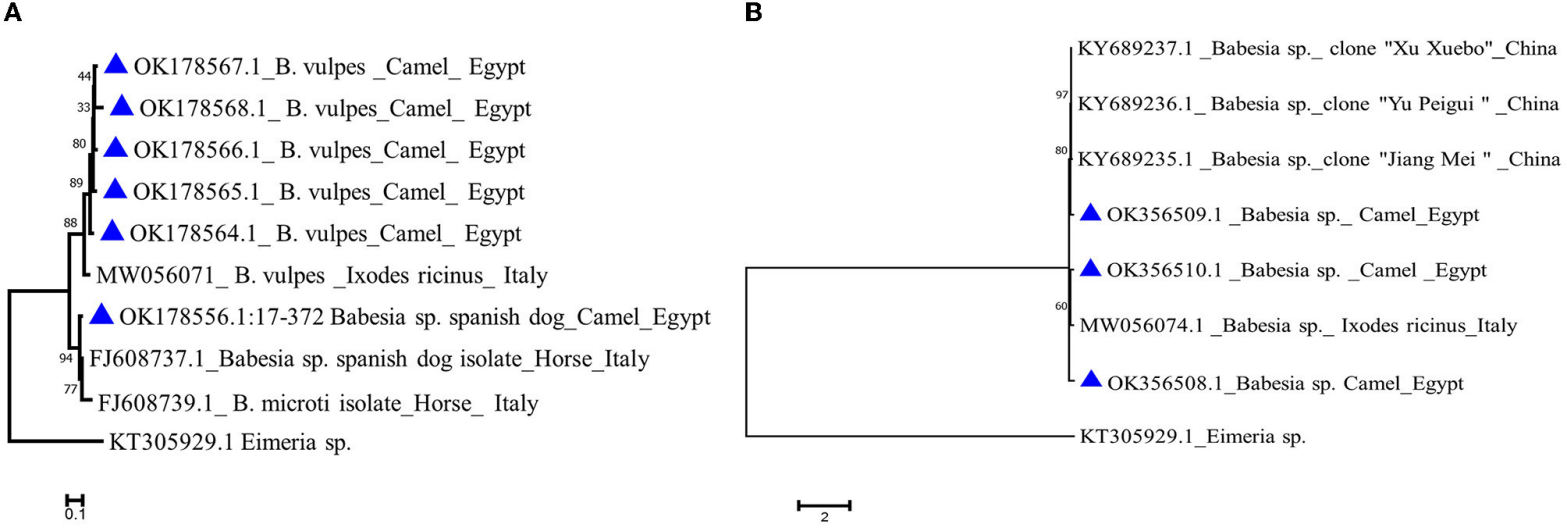
Phylogenetic analysis using the Maximum Likelihood method **(A)** *Babesia vulpes* 18S gene of camel isolates (labeled with triangle symbol) with other reference sequences identified by blast analysis and deposited in GeneBank (accession numbers shown in the tree). **(B)** *Babesia* sp. 18S gene of camel isolates in the present study labeled with a triangle symbol with other reference sequences identified by blast analysis and deposited in GeneBank (accession numbers shown in the tree). *Eimeria sp*. COI gene is used as an outgroup. The tree was created by MEGA 7 software.

We analyzed all sequences by nBLAST derived from the nPCR reactions described in the previous section, in order to verify their identity. Sequence analysis using nBlast indicated that five sequences of *B. vulpes* “Spanish dog” 18S rRNA amplicons derived from camels with accession no. OK178564.1–OK178568.1 have an 82–93% identity with 100% query coverage to previously published sequences of an Italian isolate from tick origin (MW056071.1), while one sequence with accession no. OK178556.1 showed a 96% identity with 100% query coverage to an Italian isolate (FJ608737.1) from horse origin (Figure 5A).

*Babesia* sp. blast analysis revealed that one isolate from camel origin with accession no. OK356509.1 showed an 84% identity with 100% query coverage to a China isolate. Another two *Babesia* sp. sequences (OK356508.1 and OK356510.1) showed an 81% identity with 99%−100% query coverage to an Italian isolate derived from a tick (MW056074.1; Figure 5B).

The nBlast analysis of *Theileria* sp. camel-derived sequence with accession no. OK356511.1 showed a 97% identity with 100% query coverage to *Theileria* sp. Italian isolate also from tick origin (MW056070.1).

## 4. Discussion

Ticks play an important role in the biological transmission of piroplasmid infection in camels and other animals. In fact, several tick species belonging to the genera *Hyalomma* and *Rhipicephalus* are known to be competent to infest camels, horses, and cattle (9, 27). In addition, mechanical transmission of blood-borne parasites could also take place at any time by biting flies, especially when camels graze in open fields where they become more exposed to vector bites (9).

Parasitic infections have seriously hampered the development of livestock production. Tick-borne hemoprotozoan parasites (*Babesia* spp. and *Theileria* spp.) are responsible for most of these infections. Given this scenario, the aim of the present cross-sectional study was the detection of the prevalence of camel piroplasm in Egypt using microscopic and molecular approaches, followed by the genetic characterization and phylogenetic analysis of the detected species. The prevalence of *Babesia* spp. in camel microscopically (11%) in the current study was similar to what was reported in previous studies in Egypt by Barghash et al. (1) at 11.8%. However, it was higher than that reported by Abdel Gawad (29) (4.7%) and lower than that recorded by Abd El Maleck et al. (16) (54%). Varied microscopical prevalence of *Babesia* spp. was recorded in other countries such as Saudi Arabia (13%) (30), Iraq [10%, 53%, and 17.5% by Jasem et al. (9), AL-Naily (31), and Ali and Abd (4), respectively], and Iran (0.6%) (32). The microscopical examination is a simple diagnostic method but may frequently result in false negatives in the case of carrier animals, where the parasitemia is very low. In addition, microscopic examinations alone cannot be used to accurately identify the species of *Piroplasma* with identical sizes or morphologies (1). Thus, clearly, an effective diagnosis of parasitic infections requires highly sensitive and specific tests that can identify the parasites and distinguish between species and subspecies (33). Therefore, in this study, we used PCR-based methods, followed by sequencing, in order to accurately estimate the prevalence and identification of *Piroplasma* spp. in camels in several locations in Egypt.

The molecular diagnostic results emerging from the cPCR analysis revealed that 38% of the camel blood samples analyzed in this study were infected with *Babesia/Theileria* spp. As it can be expected, the prevalence of camel piroplasm, estimated using a molecular method (38%), is specific and significantly higher than the microscopical prevalence (11%). This difference can be attributed to the low sensitivity of the microscopic examination when compared with PCR techniques (9).

The mPCR result confirmed recent studies (13) showing that camels can be infected with at least four distinct piroplasmid spp. (*T. equi, B. caballi, B. bovis*, and *B. bigemina*), with *T. equi* as the most prevalent agent. In addition, the results reported hereby represent the first molecular report of *B. caballi* infections and the first genetic characterization of *B. bovis* CPSII and *B. bigemina* in camels in Egypt. The presence of diverse *Piroplasma* spp. in camels is possibly due to the movement of animals among different locations. In addition, the processes involved in trading the herds, either by buying or selling animals (8), may result in contact and interactions with ticks derived from infected equines or bovines. Moreover, dogs may play important roles as parasite reservoirs and as hosts for transmission-competent ticks, which ultimately may result in the transmission of *B. caballi* and *T. equi* to camels via competent ticks. This possibility is supported by the detection of equine piroplasms in dogs (34, 35).

The mPCR results also confirmed that the prevalence of *T. equi* (41%) is much higher than that of *B. caballi* (5.4%), in contrast with studies by Qablan et al. (8) in Jordan, reporting that the incidence of *B. caballi* (60%) was higher than *T. equi* (40%) of positive samples. In addition, our results differ from the data reported by Jasem et al. (9) in Iraq, which also showed a higher incidence of *B*. *caballi* (39.5%) compared to *T. equi* (23.7%). Moreover, we found that the prevalence of *B. bovis* (4%) is higher than *B. bigemina* (0.5%). However, this trend agrees with data reported in previous studies performed on camel blood samples in Egypt: Barghash et al. (10) (*B. bovis*, 59.1% and *B. bigemina*, 40.9%), Salman et al. (13) (*B. bovis*, 9.6% vs. *B. bigemina*, 14.6%), and Mostafa and Dajem (36) in Saudi Arabia *(B. bovis*, 6.25% and *B. bigemina*, 0%). In contrast, Al-Naily (31) reported the opposite in Iraq (*B. bigemina*, 12.2% and *B. bovis*, 8.9%). It is likely that the differences in prevalence estimations among all these studies may be influenced by several factors, including the use of tick control methods, climatic, ecological, and environmental factors, the timing of the sample collections, the number of examined animals, nutritional and health status of the animals, animal management, the presence of parasite reservoirs, and the distinct analytical methods used, among other possibilities.

The results of the current study indicate the occurrence of variation between different camel *T. equi* and *B. caballi* sequences in Egypt. This might be since most of the camels in Egypt were imported from different countries, such as Sudan and Ethiopia.

Blast analysis of the obtained sequences revealed the presence of three new species of piroplasm infecting camel for the first time: *B. vulpes, Babesia* sp., and *Theileria* sp. *Babesia vulpes* is a canine piroplasmid (37). It was shown to be a member of the *Babesia* group infecting carnivores and is also closely related to the *B. microti* group. Subsequently, it was reclassified as *B. vulpes* (38), replacing the previous denominations as *T. annae, B. annae, B. micoti, B. microti-*like piroplasm, and *Babesia* sp. Spanish dog (39). It was also found that *B. vulpes* is closely related to *B. micoti* but exclusively can infect carnivores. In addition, and in contrast to *B. microti, B. vulpes* is not considered to be a zoonotic species. Its typical hosts were found to be a red fox, gray fox, and golden jackal, and it was detected so far in Portugal, Austria, France, Germany, Israel, Italy, Spain, and Turkey (39). The mode of transmission and tick vector of *B. vulpes* have not been determined yet. However, it was reported that dog-to-dog transmission of *B. vulpes* may be a frequent mode of transmission (39). It is more frequent in foxes than dogs (39) but was also detected in cats from Portugal (27) and Italy (40). Clinicopathologic data in *B. vulpes*-infected dogs, both with and without co-infections, included anemia, thrombocytopenia, hyperglobulinemia, hypoalbuminemia, and proteinuria (41). To the best of our knowledge, this is the first report of *B. vulpes* in camels, and the first report of this parasite in the African continent. In addition, in the present study, we report the detection of DNA derived from a newly unidentified species of *Babesia*. Interestingly, this unidentified parasite was previously detected in black bears from Japan (*Babesia* sp. lwate AB586027.1) and in giant pandas in China (42), indicating that this species may be a novel sp., currently named *Babesia* sp. EBP01. In addition, a *Theileria* sp., which was identified in camel in the present study, was previously detected in cattle and resembled *T. annulata* (43).

Altogether, the data, in addition to other recent similar reports (13), suggest relatively high levels of incidence of babesiosis and theileriosis in camels in Egypt. However, it remains unclear how these infections may affect camels' performance and reproductive ability, and whether such infected camels may act as reservoirs for *Babesia* and *Theileria* parasites that may compromise cattle, ovine, or other susceptible vertebrate species of economic importance. These data should help guide the need, design, and implementation of control measures. More research is necessary to identify all possible agents of piroplasmosis in camels in Egypt and to shed light on important health and epidemiological issues that may limit important food resources in this country.

## 5. Conclusion

Hereby, we report a high incidence of *T. equi, B. caballi, B. bigemina, B. bovis, B. vulpes, Babesia* sp., and *Theileria* sp. infections using a relatively large and diverse sample of camels in Egypt using mPCR and nPCR followed by sequencing. The molecular diagnosis followed by sequencing and genetic characterization is more sensitive and specific than ME and PCR only. This study represents the first report on the presence of *B. vulpes* in camels. Further investigations are required to determine other different *Piroplasma* spp. that might infect camels.

## Data availability statement

The datasets presented in this study can be found in online repositories. The names of the repository/repositories and accession number(s) can be found in the article/Supplementary material.

## Ethics statement

The animal study was reviewed and approved by Egyptian Drug Authority (EDA) and Ministry of Health and Population (MOHP) and the Institutional Animal Care and Use Committee (IACUC) (21277082021).

## Author contributions

AN, MM, OM, BE, and HA: methodology. BE, HA, and MM: software, investigation, and writing—original draft preparation. BE: validation. AN, OM, MM, and BE: formal analysis. MM, HA, OM, and BE: resources. CS, HA, OK, and BE: data curation. BE, MM, OM, and AN: writing review and editing. CS, AN, BE, OM, MM, OK, and HA: visualization. CS, AN, MM, OM, OK, and BE: supervision. OM, AN, MM, OK, and HA. All authors contributed to the article and approved the submitted version.

## Supplementary material

Supplementary Figure 1 Phylogenetic analysis using the Maximum Likelihood method of **(A)** *T. equi* 18S rRNA gene of camel isolates in the present study labeled with a triangle symbol with other reference sequences of *T. equi* 18S. **(B)** *B. caballi* 18S rRNA gene of camel isolates in the present study labeled with a triangle symbol with other *B. caballi* 18S reference sequences accession numbers. **(C)** *B. bigemina* 18S rRNA gene of camel isolates in the present study labeled with a triangle symbol with other *B. bigemina*18S reference sequence accession numbers. *Eimeria sp*. is used as an outgroup. This tree was created by MEGA 7 software.

Supplementary Figure 2 Phylogenetic analysis using the Maximum Likelihood method of *B. bovis* CPSII gene of camel isolates in the present study labeled with a triangle symbol with other *B. bovis* CPSII gene reference sequences accession numbers created by MEGA 7 software. *Eimeria sp*. is used as an outgroup.

Supplementary Table 1 Identity percent of *T. equi* 18S rRNA gene sequences derived from camel isolates from Egypt with *T. equi* 18S rRNA sequences from other *T. equi* 18S rRNA derived from distinct geographic isolates deposited in GenBank.

Supplementary Table 2 Identity percentages of the *B. caballi* 18S rRNA sequences derived from camel isolates with 18S rRNA sequences from other 18S rRNA sequences derived from distinct *B. caballi* geographic isolates deposited in GenBank.

Supplementary Table 3 Identity percentages of the *B. bigemina* 18S rRNA sequences derived from camel isolates with 18S rRNA sequences from other *B. bigemina* 18S rRNA sequences derived from distinct geographic isolates deposited in GenBank.

Supplementary Table 4 Identity percentages of the *B. bovis* 18S rRNA sequences derived from camel isolates with 18S rRNA sequences from other *B. bovis* 18S rRNA sequences derived from distinct geographic isolates deposited in GenBank.
