# Supplementary figures and images for: Cross-sectional analysis of Piroplasma species-infecting camel (Camelus dromedaries) in Egypt using a multipronged molecular diagnostic approach
Source: Front Vet Sci. 2023 Apr 28;10:1178511. doi: 10.3389/fvets.2023.1178511 (PMC10175621; doi:10.3389/fvets.2023.1178511)

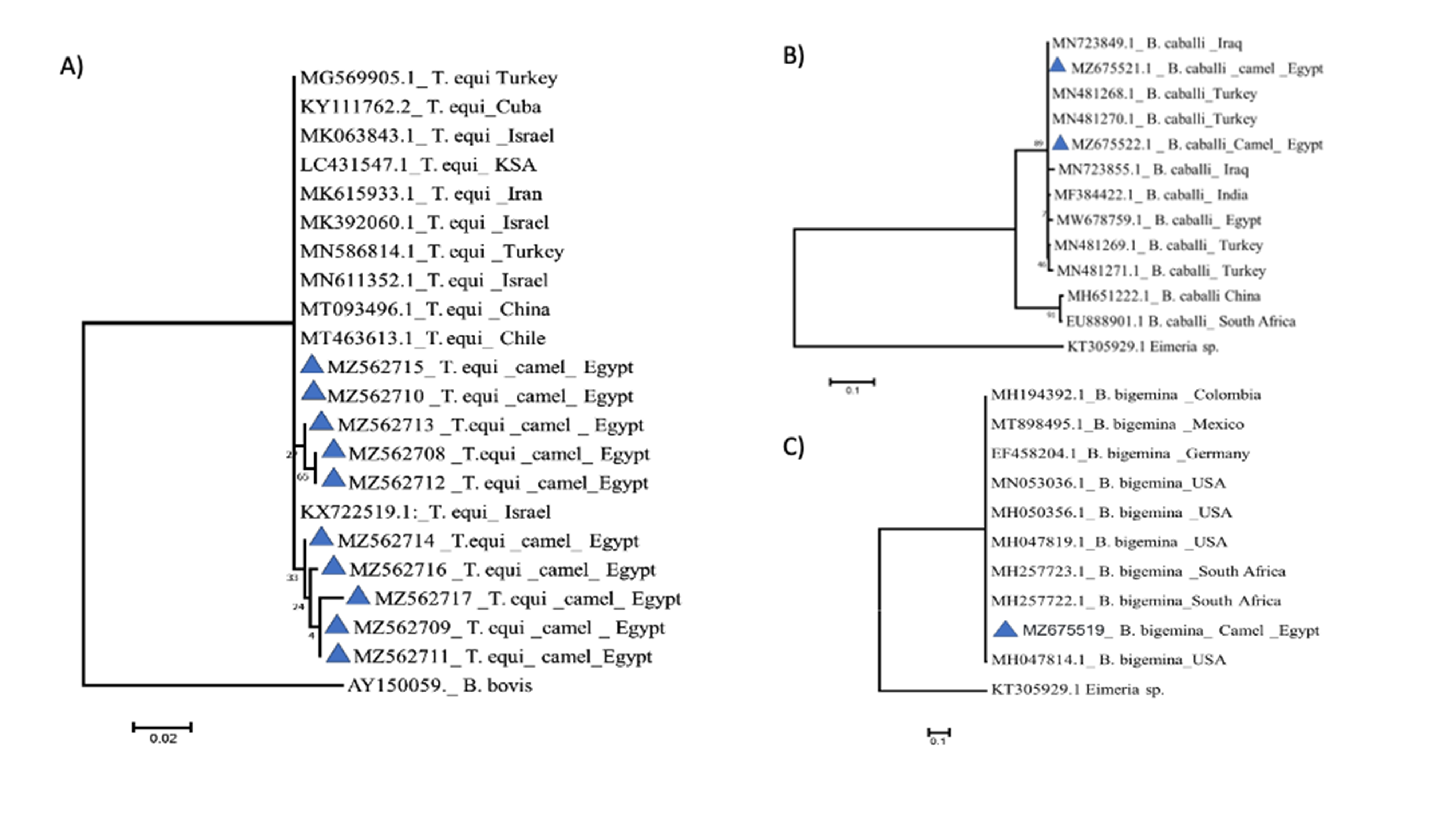

Supplement: Supplementary Figure 1 — Phylogenetic analysis using the Maximum Likelihood method of (A) T. equi 18S rRNA gene of camel isolates in the present study labeled with a triangle symbol with other reference sequences of T. equi 18S. (B) B. caballi 18S rRNA gene of camel isolates in the present study labeled with a triangle symbol with other B. caballi 18S reference sequences accession numbers. (C) B. bigemina 18S rRNA gene of camel isolates in the present study labeled with a triangle symbol with other B. bigemina18S reference sequence accession numbers. Eimeria sp. is used as an outgroup. This tree was created by MEGA 7 software. [file Image_1.TIFF]

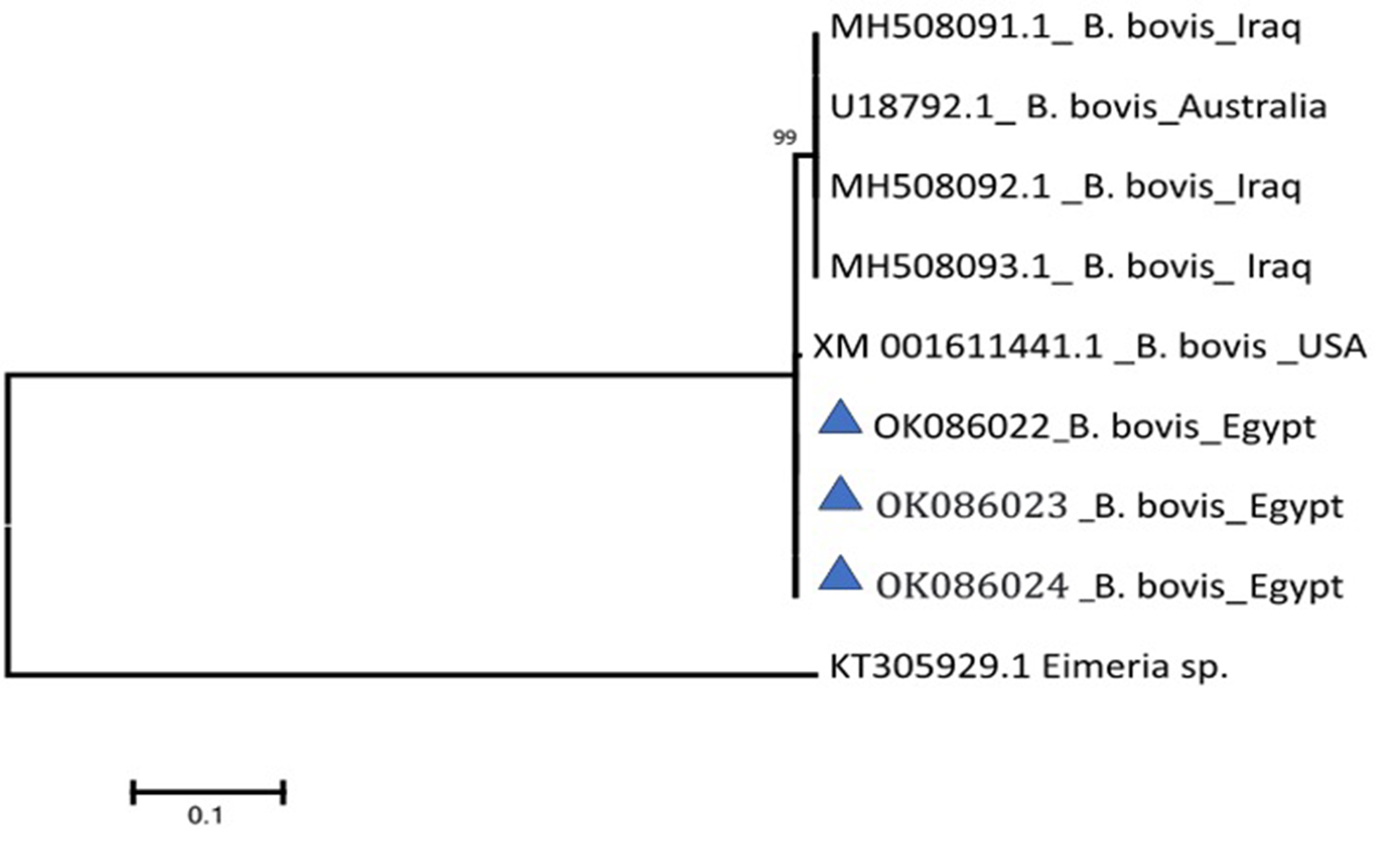

Supplement: Supplementary Figure 2 — Phylogenetic analysis using the Maximum Likelihood method of B. bovis CPSII gene of camel isolates in the present study labeled with a triangle symbol with other B. bovis CPSII gene reference sequences accession numbers created by MEGA 7 software. Eimeria sp. is used as an outgroup. [file Image_2.JPEG]
