# Supplementary material for: Cross-sectional analysis of Piroplasma species-infecting camel (Camelus dromedaries) in Egypt using a multipronged molecular diagnostic approach
Source: Front Vet Sci. 2023 Apr 28;10:1178511. doi: 10.3389/fvets.2023.1178511 (PMC10175621; doi:10.3389/fvets.2023.1178511)
Supplement: Supplementary Table 1 — Identity percent of T. equi 18S rRNA gene sequences derived from camel isolates from Egypt with T. equi 18S rRNA sequences from other T. equi 18S rRNA derived from distinct geographic isolates deposited in GenBank. [file Table_1.DOCX]

**Table (1). Identity percent of *T. equi* 18S rRNA gene sequences derived from camel isolates from Egypt with *T. equi*** **18S rRNA sequences from other *T. equi* 18S rRNA derived from distinct geographic isolates deposited in GenBank**

| Accession no. | MT463613.1 Chile  (Identity %) | MT093496.1 China (Identity %) | MN611352.1 Israel  (Identity %) |
| --- | --- | --- | --- |
| MZ562708.1_camel_Egypt | 98.7% | 98.7% | 98.7% |
| MZ562709.1_camel_Egypt | 99% | 99% | 99% |
| MZ562710.1_camel_Egypt | 100% | 100% | 100% |
| MZ562711.1_camel_Egypt | 99.3% | 99.3% | 99.3% |
| MZ562712.1_camel_Egypt | 98.9% | 98.9% | 98.9% |
| MZ562713.1_camel_Egypt | 99.6% | 99.6% | 99.6% |
| MZ562714.1_camel_Egypt | 99.6% | 99.6% | 99.6% |
| MZ562715.1_camel_Egypt | 99.6% | 99.6% | 99.6% |
| MZ562716.1_camel_Egypt | 99.2% | 99.2% | 99.2% |
| MZ562717.1_camel_Egypt | 98% | 98% | 98% |

**Table (2).** **Identity percentages of the *B. caballi* 18S rRNA sequences derived from camel isolates with 18S rRNA sequences from other 18S rRNA sequences derived from distinct *B. caballi* geographic isolates deposited in GenBank**

| Accession no | MN723849.1 Iraq  (Identity %) | MN481269.1 Turkey  (Identity %) | MF384422.1 India  (Identity %) |
| --- | --- | --- | --- |
| MZ675521.1_camel_Egypt | 99.9% | 99.6% | 99.7% |
| MZ675522.1_camel_Egypt | 100% | 99.5% | 99.8% |

**Table (3).** **Identity percentages of the *B. bigemina* 18S rRNA sequences derived from camel isolates with 18S rRNA sequences from other *B. bigemina* 18S rRNA sequences derived from distinct geographic isolates deposited in GenBank.**

| Accession no. | MH050356.1  USA  Identity % | MH257718.1South Africa  Identity % | MH194392.1  Colombia  Identity % |
| --- | --- | --- | --- |
| MZ675519.1_camel_Egypt | 100% | 100% | 100% |

**Table (4). Identity percentages of the *B. bovis* 18S rRNA sequences derived from camel isolates with 18S rRNA sequences from other *B. bovis* 18S rRNA sequences derived from distinct geographic isolates deposited in GenBank**

| Accession no. | MH508093.1 Iraq  Identity % | MH508092.1  Iraq  Identity % | MH508091.1 Iraq  Identity % |
| --- | --- | --- | --- |
| OK086022.1_camel_Egypt | 97.5% | 97.5% | 97.5% |
| OK086023.1_camel_Egypt | 97.1 | 97.1% | 97.1 |
| OK086024.1_camel_Egypt | 97.4% | 97.4% | 97.4% |
